# Blood pressure and heart failure: focused on treatment

**DOI:** 10.1186/s40885-024-00271-y

**Published:** 2024-06-01

**Authors:** Kyeong-Hyeon Chun, Seok-Min Kang

**Affiliations:** 1 Division of Cardiology, National Health Insurance Service Ilsan Hospital, Goyang, Republic of Korea; 2 Division of Cardiology, Severance Hospital, Cardiovascular Research Institute, Yonsei University College of Medicine, Seoul, Republic of Korea

**Keywords:** Blood pressure, Hypertension, Heart failure

## Abstract

Heart failure (HF) remains a significant global health burden, and hypertension is known to be the primary contributor to its development. Although aggressive hypertension treatment can prevent heart changes in at-risk patients, determining the optimal blood pressure (BP) targets in cases diagnosed with HF is challenging owing to insufficient evidence. Notably, hypertension is more strongly associated with HF with preserved ejection fraction than with HF with reduced ejection fraction. Patients with acute hypertensive HF exhibit sudden symptoms of acute HF, especially those manifested with severely high BP; however, no specific vasodilator therapy has proven beneficial for this type of acute HF. Since the majority of medications used to treat HF contribute to lowering BP, and BP remains one of the most important hemodynamic markers, targeted BP management is very concerned in treatment strategies. However, no concrete guidelines exist, prompting a trend towards optimizing therapies to within tolerable ranges, rather than setting explicit BP goals. This review discusses the connection between BP and HF, explores its pathophysiology through clinical studies, and addresses its clinical significance and treatment targets.

## Background

Heart failure (HF) remains a major public health burden, with a rapidly increasing global prevalence. In the United States, more than 5 million people aged ≥20 years are affected by HF [1], and this number is expected to increase by 46%, resulting in an estimated 8 million Americans with HF in 2030 [2]. Hypertension is one of the most frequent comorbidities [3], playing a pivotal role in the development of HF [4]. In the Framingham Heart Study, hypertension progressed to HF in 91% of patients > 20 years of age, with a doubling and tripling of the risk of HF in male and female hypertensive patients, respectively [5, 6]. Chronic hypertension causes functional and structural changes in the heart, culminating in HF and further increasing the rate of mortality and morbidity [7]. Intensive treatment of hypertension can prevent and reverse myocardial changes in patients at risk of HF; however, defining optimal blood pressure (BP) targets for patients who have already developed HF is challenging owing to a lack of evidence.

Currently, HF is classified depending on the left ventricular ejection fraction (LVEF), with LVEF ≤40% defined as HF with reduced ejection fraction (HFrEF) and LVEF ≥50% as HF with preserved ejection fraction (HFpEF) [8]. In addition, if the LVEF is between 41 and 49%, the definition of HF with mildly reduced ejection fraction (HFmrEF) is commonly used in a dynamic trajectory to denote improvement from or deterioration to HFrEF [9]. HFmrEF occupies a spectrum between HFrEF and HFpEF, exhibiting the characteristics of both. However, this classification system is often ambiguous [10]. In the present review, rather than focusing on this detailed classification of LVEF, we focus on the classical phenotypes of HFrEF and HFpEF (implicitly including the concept of HFmrEF) and their association with BP from a more comprehensive perspective.

### Development of hypertensive heart disease and HF

Traditionally, the development and progression of HF in hypertensive patients has been classified into four stages: (1) isolated left ventricular (LV) diastolic dysfunction without LV hypertrophy; (2) LV diastolic dysfunction with concentric LV hypertrophy; (3) clinical HFpEF accompanied by pulmonary edema; and (4) dilated cardiac chambers with HFrEF [6]. These stages suggest that diastolic dysfunction is an early phenomenon, and hypertension-induced LV hypertrophy leads to remodeling of the left atrium and ventricle, ultimately resulting in advanced diastolic and systolic dysfunction.

As reviewed in the article by Messerli et al. [6], hypertensive heart disease plays a pivotal role in the pathophysiology of HF through a sequential and intricate process. Initially, the LV responds to elevated BP by adapting to the hemodynamic wall stress, which results in pressure overload. This adaptation involves the thickening of the LV wall and an increase in LV mass, resulting in concentric LV hypertrophy. During this phase, the initial manifestation of cardiac dysfunction is LV diastolic dysfunction. With a persistent pressure overload, diastolic dysfunction progresses, ultimately leading to the onset of HFpEF. In the advanced stages of hypertensive heart disease, typically due to prolonged exposure to pressure overload with or without concurrent myocardial ischemia, the condition evolves into a dilated LV dimension. The final stage is characterized by reduced LVEF and development of HFrEF.

However, HFrEF and HFpEF should be considered from a slightly different perspective than that presented by Messerli et al. [6]. If HFrEF and HFpEF are considered part of a unified disease spectrum, they may be expected to respond similarly to HF treatment. However, numerous medications that have demonstrated clear improvements in HFrEF have not shown comparable beneficial effects on HFpEF [11]. Angiotensin receptor blockers (ARBs) [12], angiotensin-converting enzyme inhibitors (ACEis) [13], β-blockers [14, 15], and mineralocorticoid receptor antagonists (MRAs) [16], all of which are also used as antihypertensive drugs, have failed to show clinically significant prognostic improvements in HFpEF, unlike in HFrEF. These differences were also evident in epidemiological studies. For example, a Japanese chronic HF registry-based study showed that most patients with HFpEF and nearly half of those with HFrEF remained in their respective categories throughout a 3-year follow-up period [10]. A study consisting of 3480 consecutive Japanese patients with HF showed that HFpEF transitioned to HFrEF in only 4% of them after 3 years, whereas HFrEF at registration transitioned to HFpEF and HFmrEF in 26 and 21% of patients, respectively, at 3 years, suggesting reverse remodeling after treatment [10]. These findings further support the idea that HFpEF and HFrEF are distinct syndromes with fundamental pathophysiological differences and etiologies. Similarly, in another long-term longitudinal study of ambulatory HFpEF patients, LVEF remained ≥50% in most patients with HFpEF for 11 years, and only 1.6% of patients evolved to LVEF < 50% [17]. Therefore, approaching HFrEF and HFpEF differently when examining their associations with hypertension is imperative.

### Association between hypertension and HF

Hypertension is widely recognized as one of the most important risk factors of HFpEF [18]. Increased LV filling pressure and chronic myocardial remodeling due to hypertension are considered the primary mechanisms leading to the development of HFpEF [19]. Elevated systolic BP (SBP) is notably observed in patients with HFpEF, with a 3% rise in the likelihood of prevalent HFpEF for every 1 mmHg increase in SBP > 120 mmHg in an acute HF setting [20].

In terms of HFrEF, the association manifests distinctively. According to the European Society of Cardiology Heart Failure Long-Term Registry, the largest pan-European cohort of patients with real-world chronic HF in the full spectrum of LVEF, HFrEF accounts for approximately 60% of all patients in the registry [21]. This registry data showed that mean SBP tends to be lower in HFrEF than in other categories, with 121.6 ± 20.8 mmHg in HFrEF, 126.5 ± 21.1 mmHg in HFmrEF, and 130.9 ± 21.4 mmHg in HFpEF. The use of antihypertensive therapy differed notably between the HFrEF and HFpEF groups, with 56% for HFrEF and 67% for HFpEF. Regarding the underlying etiology of HF, nearly half of HFrEF cases (49%) occur due to ischemic heart disease, approximately one-third (35%) is caused by idiopathic dilated cardiomyopathy, and only 4.5% is due to hypertension itself. In contrast, HFpEF has a different etiology, with 18% of cases occurring due to hypertension, approximately a quarter due to ischemic heart disease, and 12% due to idiopathic dilated cardiomyopathy.

An analysis of the Organized Program to Initiate Lifesaving Treatment in Hospitalized Patients with Heart Failure (OPTIMIZE-HF) registry based on the United States population mirrors these distinctions [14]. The etiology of HF was ischemic in a higher percentage of patients with HFrEF than in those with HFpEF (54% vs. 38%, *P* < 0.0001), whereas the hypertensive etiology was more common in patients with HFpEF than in those with HFrEF (28% vs. 17%, *P* < 0.0001). Interestingly, when further stratified by LVEF, a hypertensive etiology was significantly predominant in HFpEF (LVEF, > 50%) than in HFmrEF (LVEF, 41–49%) at a rate of 31% versus 22% (*P* < 0.0001). In the Korean Acute Heart Failure (KorAHF) registry, which is a prospective multicenter cohort registry including more than 5600 patients with acute heart failure from 10 tertiary hospitals in the Republic of Korea, the prevalence of hypertension was 62.2%; ischemic etiology accounted for 37.6%, and idiopathic dilated cardiomyopathy comprised 15.3% of the cases [22], which is in line with the European HF registry. When divided based on LVEF, the prevalence of hypertension was higher in patients with HFpEF (64%) than in those with HFrEF (56%).

These observations suggest that while some variability may be influenced by factors such as race, region, and specific registry characteristics, a stronger association exists between high BP and HFpEF than between high BP and HFrEF in the overall population with HF.

### Hypertensive AHF

Acute HF (AHF) is caused by the acute or subacute deterioration of heart function, leading to pulmonary edema and subsequent symptoms such as dyspnea or edema. Given that these symptoms are primarily caused by volume overload, treatment strategies are based on this assumption [23]. However, a closer look reveals that the aggravating factors of HF are diverse, resulting in distinct phenotypes of AHF that necessitate more specialized treatments. These phenotypes can occur as acute exacerbation of preexisting chronic HF, or as a new onset (de novo) HF. Concerning the relationship between BP and AHF, lowering the ventricular filling pressure plays a crucial role in AHF management, especially when hypertension is concurrently present [24].

AHF is a complex and multifaceted condition characterized by diverse etiologies, distinct pathophysiological mechanisms, varying risk profiles, and treatment responses [25, 26]. This heterogeneity poses significant challenges when conducting randomized controlled trials aimed at comprehensively investigating AHF. In this context, we often encounter a specific form of AHF where “high BP” is clearly the cause or is strongly suspected of contributing to the pathogenesis, which is commonly referred to as “hypertensive acute heart failure (H-AHF)”. This clinical phenomenon is characterized by a dramatic improvement in clinical signs and symptoms by BP-lowering treatment, which is also the goal of treatment. In previous studies, the H-AHF has often been defined by the following two features [23, 24, 27, 28]: (1) SBP ≥ 140 mmHg and (2) acute cardiogenic pulmonary edema, often with rapid onset.

Within the spectrum of AHF, approximately half of the patients may exhibit an SBP > 140 mmHg [29–31], although not all cases are categorized as H-AHF. H-AHF is particularly characterized by the sudden onset of symptoms, notably pulmonary edema, which distinguishes it from other forms of AHF [23, 28]. A more obvious characteristic of H-AHF is the presence of severely elevated BP (≥160–180 mmHg), with pulmonary edema developing in a matter of hours, and no other cause of AHF except hypertension [24, 31]. However, because of this vague definition and characterization, there is a large variation in prevalence between the registries; this phenotype is reported as 4% in the KorAHF registry [22] and approximately 11% in the European or US HF registries [21, 30, 31]. In particular, for HFrEF, hypertensive etiology is reported as 4.5% in the European registry [21] and 2.9% in the KorAHF registry [22]. This difference is thought to be due to demographic variations and ambiguity in the definition of diagnosis.

Several studies have investigated the association between symptom duration and the clinical features of patients with H-AHF. One study examined whether dyspnea occurred in ≤7 or > 7 days, and found that the latter was associated with higher in-hospital worsening of HF and 1-year cardiovascular mortality and less improvement in symptoms within 48 hours [32] . In the group with onset ≤7 days, SBP was significantly higher (138 mmHg vs. 121 mmHg) and moderate-to-severe pulmonary edema was more frequent (33% vs. 8%) compared to cases with onset > 7 days. Although these findings do not precisely delineate the threshold for a “rapid” onset indicative of H-AHF pathophysiology, they do provide knowledge regarding the phenotype. In other words, H-AHF may manifest as the most severe form of AHF with high BP; however, it also exhibits a relatively favorable prognosis [24, 32–36]. This is supported by studies showing that among patients with AHF presenting to the emergency department, high BP is often a predictor of low risk [27, 34–36].

A recent post hoc analysis demonstrated that treatment effectiveness varied with BP [37]. It has been recommended that SBP should be lowered by ≤25% in H-AHF [24, 27, 38]. Patients treated with vasodilators who achieved an SBP reduction ≤25% within 6 hours of emergency room arrival had a better diuretic response and lower 1-year mortality than those with SBP reduction > 25% [39]. In this regard, vasodilators are hypothesized to improve outcomes by mitigating end-organ damage in patients with H-AHF, potentially by influencing both preload and/or afterload [24], and they can generally be used safely in H-AHF and may provide benefits when applied to appropriate patients. Unfortunately, despite numerous randomized clinical trials in this population over the past two decades, no vasodilator has shown any mortality benefit [40]. This is due to the fact that AHF is a heterogeneous condition with diverse etiologies and pathophysiology, and stratifying and enrolling specific subgroups with predictable treatment responses is challenging. Although there is a lack of evidence from randomized clinical trials, intravenous nitroglycerin, which is still the most familiar vasodilative agent among clinicians, can be administered safely and effectively to improve outcomes in patients with AHF and severely high BP [41].

### Prognostic value of BP in HF

We recognize that there is no alternative to BP measurement as a source of clinical information regarding the hemodynamic status of patients with HF. Indeed, owing to its simplicity in measurement and widespread availability, BP is of paramount clinical importance in guiding the treatment of patients with HF. Furthermore, arterial hypertension is considered one of the most common comorbidities [3] and a precursor of HF [4]. Table 1 [42–57] shows the previous clinical trials and observational studies on the prognosis of BP in patients with and without HF.

**Table 1:** Summary of clinical trials and observational studies on prognosis with respect to BP in subjects with and without HF

Study	Participants	Methods/intervention	Clinical endpoint	Association of BP and outcomes
RCT for patients with HF				
Rouleau et al. [42] (COPERNICUS study, 2004)	2289 Patients with chronic HF (LVEF < 25%)	1:1 Randomized trial (carvedilol vs. placebo)	Primary endpoint: all-cause mortality	Lowest SBP had the highest risk of event
Lee et al. [43] (2006)^a^	5747 Patients with HF (LVEF < 45%)	1:1 Randomized trial (digoxin vs. placebo)	Primary endpoint: all-cause mortality	SBP < 100 mmHg had a higher risk of mortality compared to SBP of 130–139 mmHg (HR, 1.65; 95% CI, 1.25–2.17)
Desai et al. [44] (2010)^b^	2706 Patients with chronic HF, LVEF ≤35%, NYHA III or IV	1:1 Randomized trial (bucindolol vs. placebo)	Primary endpoint: all-cause mortality Secondary endpoint: HF readmission	SBP ≤120 mmHg had a higher risk of HF readmission compared to SBP > 120 mmHg, but had no association with all-cause mortality
Banach et al. [45] (2011)^a^	7785 Patients with chronic systolic and diastolic HF (NYHA I–III; mean LVEF, 29%)	1:1 Randomized trial (digoxin vs. placebo)	Primary endpoint: all-cause mortality Secondary endpoint: cause-specific mortality and readmission	SBP ≤120 mmHg had a higher risk of CV mortality and all-cause, CV, and HF readmission compared to SBP > 120 mmHg, but had no association with all-cause mortality
Böhm et al. [46] (PARADIGM-HF trial, 2017)	8442 Patients with HFrEF (LVEF, ≤40%)	1:1 Randomized trial (sacubitril/valsartan vs. enalapril)	Primary endpoint: a composite of death from cardiovascular causes or hospitalization for HF Secondary endpoint: cardiovascular death, HF hospitalization, and all-cause death	Compared to baseline SBP < 110 mmHg group, risk for all outcomes was lower with higher SBP, although less clearly in patients with SBP ≥140 mmHg
Selvaraj et al. [47] (PARAGON-HF trial, 2019)	4795 Patients with HFpEF (LVEF, ≥45%)	1:1 Randomized trial (sacubitril/valsartan vs. valsartan)	Primary endpoint: cardiovascular death and total (first and recurrent) HF hospitalizations	Baseline and mean achieved SBP of 120–129 mmHg had the lowest risk of primary endpoint Baseline SBP did not modify the effect of sacubitril/valsartan, and its BP-lowering effects were not related to its effects on outcomes
Lam et al. [48] (2021)^c^	5050 Patients with chronic HF (NYHA II–IV; LVEF, < 45%) Subgroup: > 75 yr, baseline SBP 100–110 mmHg, and taking ARNI	1:1 Randomized trial (vericiguat vs. placebo)	Primary endpoint: a composite of death from cardiovascular causes or first hospitalization for HF Safety endpoint: occurrence of symptomatic hypotension or syncope	Safety event rates were similar between both arms within each subgroup Primary endpoint was similar across the spectrum of baseline SBP
Observational study for patients with HF				
Vidán et al. [49] (National Heart Failure project, 2010)	56,942 Patients aged ≥65 yr hospitalized for HF	Observational study	Primary endpoint: all-cause mortality	Higher SBP on admission was significantly associated with lower 30-day and 1-year mortality Using SBP 120–149 mmHg as the reference, odds ratio for 1-year mortality were 2.18 for SBP < 90 mmHg and 1.57 for SBP 90–119 mmHg
Lee et al. [50] (2017)^d^	4487 Patients hospitalized for acute HF (mean LVEF, 38%)	Observational study (on-treatment BP data available for patients)	Primary endpoint: all-cause mortality	A nadir of 132.4/74.2 mmHg had the lowest mortality SBP and DBP had a relationship with increased mortality above and below the reference BP In HFrEF, only lower BP was related with increased mortality
Arundel et al. [51] (2019)^e^	10,535 Patients with HFrEF (LVEF, ≤40%)	Observational study (propensity-matched cohort analysis)	Primary endpoint: all-cause mortality Secondary endpoint: all-cause readmission and HF readmission	Compared to SBP ≥130 mmHg, SBP < 130 mmHg had a higher risk of mortality, all-cause readmission, and HF readmission
Faselis et al. [52] (2021)^e^	6778 Hospitalized HFpEF patients with hypertension	Observational study	Primary endpoint: all-cause mortality	Compared to SBP ≥130 mmHg, SBP < 130 mmHg had no association with outcomes, but SBP < 120 mmHg had a higher risk of death
Normal or hypertensive population without HF				
D’Agostino et al. [53] (Framingham study, 1991)	5209 Cohort subjects (age 30–62 yr, normal population)	Longitudinal cohort study	Primary endpoint: death from coronary arterial disease	DBP had a U-shaped relation with death from coronary arterial disease (SBP was not significant)
Ekundayo et al. [54] (2009)	5795 Cohort subjects (age ≥ 65 yr, normal population)	Longitudinal cohort study (propensity-matched cohort analysis)	Primary endpoint: new onset HF	Isolated systolic hypertension (SBP ≥140 mmHg) was associated with increased risk of incident HF, but not with all-cause mortality
Bangalore et al. [55] (2010)^f^	10,001 Patients with coronary heart disease	1:1 Randomized trial (atorvastatin 10 mg vs. 80 mg per day)	Primary endpoint: a composite of death from coronary heart disease, nonfatal, nonprocedure-related myocardial infarction, resuscitation after cardiac arrest, or stroke	A nadir of 146.3/81.4 mmHg had the lowest event rate SBP or DBP and primary outcome had a J-curve relationship with increased risk above and below the reference BP range
Kjeldsen et al. [56] (VALUE trial, 2016)	15,244 Hypertensive patients (45% had a history of coronary arterial disease)	1:1 Randomized trial (valsartan 80 mg vs. amlodipine 5 mg daily)	Primary endpoint: time to first cardiac event (composite of MI, sudden cardiac death, death from revascularization procedure, HF hospitalization, and emergency procedures to prevent MI)	SBP ≥150 mmHg had a higher risk of endpoint compared to SBP of 130–149 mmHg (SBP < 130 mmHg vs. 130–149 mmHg was not significant)
Upadhya et al. [57] (2017)^g^	9361 Hypertensive patients with an increased risk of cardiovascular disease	1:1 Randomized trial (intensive group, target SBP < 120 mmHg vs. standard group, target SBP < 140 mmHg)	Primary endpoint: acute decompensated HF (hospitalization or emergency department visit requiring intravenous diuretic or inotropic agents)	SBP target < 120 mmHg was significantly associated with a lower risk of acute decompensated HF events compared to target SBP < 140 mmHg

*BP* blood pressure, *HF* heart failure, *RCT* randomized controlled trial, *COPERNICUS* Carvedilol Prospective Randomized Cumulative Survival, *LVEF* left ventricular ejection fraction, *SBP* systolic blood pressure, *HR* hazard ratio, *CI* confidence interval, *NYHA* New York Heart Association, *CV* cardiovascular, *PARADIGM-HF* Prospective Comparison of ARNI with ACEi to Determine Impact on Global Mortality and Morbidity in Heart Failure, *HFrEF* Heart failure with reduced ejection fraction, *PARAGON-HF* Prospective Comparison of ARNI With ARB Global Outcomes in HF with Preserved Ejection Fraction, *HFpEF* heart failure with preserved ejection fraction, *ARNI* angiotensin receptor/neprilysin inhibitor, *DBP* diastolic blood pressure, *VALUE* Valsartan Antihypertensive Long-term Use Evaluation, *MI* myocardial infarction

^a^A post hoc analysis of Digitalis Investigation Group (DIG) trial. ^b^A post hoc analysis of Beta-Blocker Evaluation of Survival Trial (BEST) trial. ^c^Subgroup analysis of Vericiguat Global Study in Subjects with Heart Failure with Reduced Ejection Fraction (VICTORIA) trial. ^d^Korean Acute Heart Failure (KorAHF) registry-based study. ^e^Organized Program to Initiate Lifesaving Treatment in Hospitalized Patients with Heart Failure (OPTIMIZE-HF) registry-based study. ^f^A post hoc analysis of Treating to New Targets (TNT) trial. ^g^A post hoc analysis of Systolic Blood Pressure Intervention Trial (SPRINT) trial

In general, associating a higher BP with a greater incidence of HF is reasonable. In a population-based longitudinal observational study including 5888 adults aged ≥65 years, isolated systolic hypertension (SBP ≥140 mmHg) was associated with an increased risk of incident HF compared to subjects without isolated systolic hypertension during a follow-up duration of 8.7 years [54]. Regarding the clinical prognosis of low versus high BP, the Valsartan Antihypertensive Long-term Use Evaluation (VALUE) trial, which enrolled a high-risk population of 15,244 hypertensive patients, showed no evidence for an increased risk of adverse outcome in patients with low BP [56]. This observation holds true for hypertensive patients in general and for those at high risk of cardiovascular disease without a history of HF. However, in patients who have already been diagnosed with HF, the clinical significance of BP appears to differ from that in the general population or in those with other cardiovascular diseases.

A retrospective longitudinal study showed that a low SBP (< 90 mmHg) was associated with poor survival in patients with chronic HF [21]. Notably, when the subjects were categorized based on SBP levels (< 90, 90–109, 110–129, and > 129 mmHg), as BP increased, the prognosis tended to improve in the group with SBP > 129 mmHg. Interestingly, this study also showed that pronounced long-term changes in SBP were associated with poor prognosis in this population. This result is in line with a previous study, which suggested the concept of “reverse epidemiology” that implies an improved survival rate in patients with HF with an elevated BP [58]. Several studies have reported a similar association, and this correlation is reminiscent of the “obesity paradox,” the relationship between HF and obesity. A post hoc analysis of OPTIMIZE-HF registry showed that, compared to SBP ≥ 130 mmHg at discharge, SBP < 130 mmHg was not associated with outcomes, but SBP < 120 mmHg at discharge was associated with a higher risk of death among hospitalized elderly HFpEF patients with hypertension [52]. Recent observational studies have also indicated that low SBP is associated with poor prognosis in patients with HFpEF [59, 60].

It is not surprising that low BP might be considered harmful, as it can serve as a marker of worse health conditions. Even among patients on maximal guideline-directed medication therapy (GDMT), those with SBP < 110 mmHg have been shown to be at increased risk of readmission for HF [44], and this association remained significant despite no evidence of more severe disease or a greater burden of comorbidities in those with low BP [46].

**Fig. 1:**
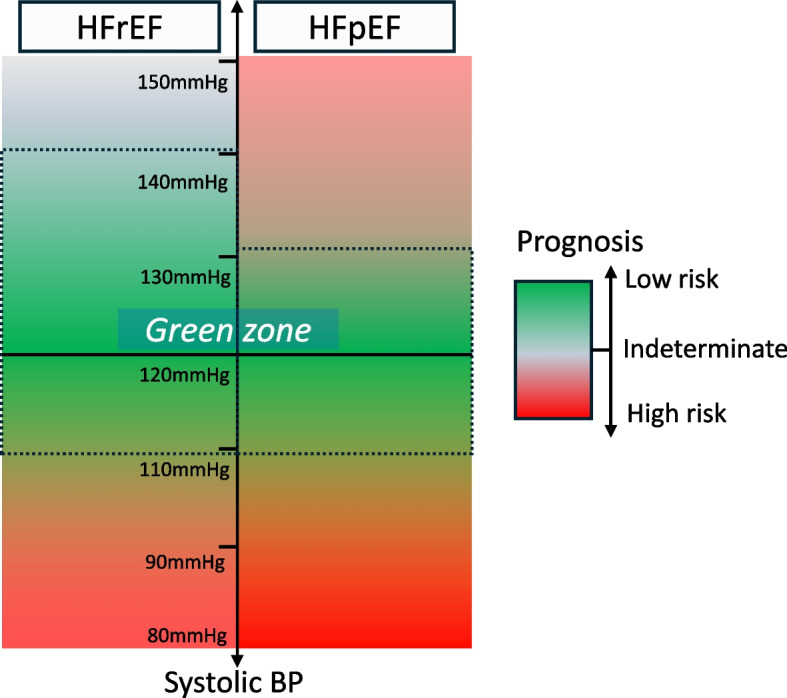
A conceptual safety margin (“green zone”) for blood pressure (BP) in each heart failure group. Heart failure with reduced ejection fraction (HFrEF) has a wide safety margin for BP, with a lower BP being at higher risk. Heart failure with preserved ejection fraction (HFpEF) has a relatively narrow safety margin for BP, with both higher and lower BP being at higher risk

While most of these data analyzed prognosis based on baseline BP, the analysis from the KorAHF registry focused on on-treatment BP during follow-up [50]. Among the 4487 patients hospitalized for acute HF, SBP and diastolic BP (DBP) above and below the reference BP were associated with increased mortality. A nadir of 132.4/74.2 mmHg was associated with the lowest mortality rate in this cohort, especially for those with HFpEF. However, in patients with HFrEF, the mortality risk increased significantly only in the lower BP range and not in the higher BP range. In detail, the lowest risk of mortality was observed at an SBP/DBP of 136.0/76.6 mmHg for HFrEF, and at 127.9/72.7 mmHg for HFpEF. This pattern of association with BP profile was also described in a previous study [61], although the classification of HF was comparatively different; patients with mild-to-moderate LV systolic dysfunction (LVEF, 30–50%) had a U-shaped association with mortality, but patients with severe LV systolic dysfunction (LVEF, < 30%) had a linear relationship with lower SBP, which was associated with increased mortality. Thus, it can be inferred that the association among HFrEF, HFpEF, and BP had a relatively different pattern. Taken together, these results suggest that there may be a safer BP range in HF, although it is not clear-cut; lower BP is associated with a higher risk in HFrEF and HFpEF, and while HFrEF has a wider margin of safety for higher BP, HFpEF has a narrower margin of safety because higher BP is also associated with increased risk in HFpEF compared to that in HFrEF (Fig. 1).

### Medication affecting BP in HF

Most agents proven to have a survival benefit in HF have the potential to lower BP (such as ACEis, ARBs, β-blockers, angiotensin receptor-neprilysin inhibitors [ARNIs], MRAs, and sodium glucose cotransporter 2 [SGLT2] inhibitors) to a greater or lesser extent; however, not all BP-lowering treatments have the same beneficial effects, as summarized in Table 2 [12, 13, 16, 42, 62–78]. It is challenging to establish a direct relationship between the probability of clinical benefit and BP-lowering alone, particularly in more recent studies where the number of medications used in the study population was higher than that in previous studies. Additionally, in some cases, lowering the BP was neither beneficial nor detrimental, thereby complicating the assessment of contribution of BP to the benefits of GDMT in HF. These conflicting results have raised the question of whether reduction in BP is due to the positive effects of drugs with BP-lowering effects or, conversely, whether these drugs have deleterious effects that are offset by the benefits of neurohumoral regulation [79]. Nevertheless, the importance of treating BP in HF is consensually recognized in the HF and hypertension guidelines [38, 80, 81] both of which recommend drugs that have been reliably demonstrated in randomized clinical trials to improve outcomes as first-line therapy, especially for HFrEF [82].

**Table 2:** Summary of studies on principal HF drugs affecting blood pressure

Study	Intervention	Control	HF type	Mean difference in SBP change (mmHg)	Mortality	HHF	Medication
ACEi							
SOLVD [62] (1991)	Enalapril	Placebo	HFrEF	∆4.7 ↓	↓	↓	BB (8%)
PEP-CHF [13] (2006)	Perindopril	Placebo	HFpEF	∆3.0 ↓	↔	↔	BB (54%) MRA (10%)
ARB							
Val-HeFT [63] (2001)	Valsartan	Placebo	HFrEF	∆4.0 ↓	↔	↓	ACEi (93%) BB (35%)
CHARM-Added [64] (2003)	Candesartan	Placebo	HFrEF	∆4.6 ↓	↔	↓	ACEi (100%) BB (55%) MRA (17%)
CHARM-Preserved [12] (2003)	Candesartan	Placebo	HFpEF	∆6.9 ↓	↔	↔	ACEi (19%) BB (56%) MRA (12%)
I-PRESERVE [65] (2008)	Irbesartan	Placebo	HFpEF	∆3.6 ↓	↔	↔	ACEi (25%) BB (59%) MRA (15%)
ARNI							
PARADIGM-HF [66] (2014)	Sacubitril/valsartan	Enalapril	HFrEF	∆3.2 ↓	↓	↓	BB (93%) MRA (56%)
PARAGON-HF [67] (2019)	Sacubitril/valsartan	Valsartan	HFpEF	∆4.5 ↓	↔	↔	ACEi/ARB (86%) BB (80%) MRA (26%)
BB							
COPERNICUS [42, 68] (2002)	Carvedilol	Placebo	HFrEF	∆2.2 ↓	↓	↓	ACEi (97%) MRA (19%)
PRECISE [69] (1996)	Carvedilol	Placebo	HFrEF	∆5.1 ↓	↓	NA	ACEi (96%)
MERIT-HF [70] (1999)	Metoprolol	Placebo	HFrEF	∆5.6 ↑	↓	NA	ACEi/ARB (95%)
SENIORS [71] (2005)	Nebivolol	Placebo	Both	∆2.0 ↓	↔	↔	ACEi/ARB (88%) MRA (28%)
MRA							
TOPCAT [16] (2014)	Spironolactone	Placebo	HFpEF	∆2.5 ↓	↔	↓	ACEi/ARB (84%) BB (78%)
EMPHASIS-HF [72] (2011)	Eplerenone	Placebo	HFrEF	∆2.2 ↓	↓	↓	ACEi/ARB (93%) BB (87%)
SGLT2 inhibitor							
EMPEROR-Reduced [73] (2020)	Empagliflozin	Placebo	HFrEF	∆0.7 ↓	↔	↓	ACEi/ARB (70%) ARNI (19%) BB (95%) MRA (71%)
EMPEROR-Preserved [74] (2021)	Empagliflozin	Placebo	HFpEF	∆1.2 ↓	↔	↓	ACEi/ARB (81%) ARNI (2%) BB (86%) MRA (37%)
DAPA-HF [75] (2019)	Dapagliflozin	Placebo	HFrEF	∆1.3 ↓	↓	↓	ACEi/ARB (84%) ARNI (11%) BB (96%) MRA (71%)
DELIVER [76] (2022)	Dapagliflozin	Placebo	HFpEF	∆1.8 ↓	↔	↓	ACEi/ARB (73%) ARNI (5%) BB (83%) MRA (48%)
PRAISE-2 [74] (2013)	Amlodipine	Placebo	HFrEF	∆5.2 ↓	↔	↔	ACEi (99%) BB (19%)
VICTORIA [77] (2020)	Vericiguat	Placebo	HFrEF	∆Trajectory, slightly↓	↔	↓	ACEi/ARB (73%) ARNI (15%) BB (93%) MRA (70%)

*HF* heart failure, *SBP* systolic blood pressure, *HHF* hospitalization for heart failure, *ACEi* angiotensin-converting enzyme inhibitor, *SOLVD* Studies of Left Ventricular Dysfunction, *HFrEF* heart failure with reduced ejection fraction, *BB* β-blocker, *PEP-CHF* Perindopril in Elderly People with Chronic Heart Failure, *HFpEF* heart failure with preserved ejection fraction, *MRA* mineralocorticoid receptor antagonist, *ARB* angiotensin receptor blocker, *Val-HeFT* Valsartan Heart Failure Trial, *CHARM* Candesartan in Heart Failure Assessment of Reduction in Mortality and Morbidity, *I-PRESERVE* Irbesartan in Heart Failure with Preserved Ejection Fraction, *ARNI* angiotensin receptor-neprilysin inhibitor, *PARADIGM-HF* Prospective Comparison of ARNI with ACEi to Determine Impact on Global Mortality and Morbidity in Heart Failure, *PARAGON-HF* Prospective Comparison of ARNI with ARB Global Outcomes in HF With Preserved Ejection Fraction, *COPERNICUS* Carvedilol Prospective Randomized Cumulative Survival, *PRECISE* Prospective Randomized Trial of the Optimal Evaluation of Cardiac Symptoms and Revascularization, *NA* not applicable, *MERIT-HF* Metoprolol CR/XL Randomized Intervention Trial in Congestive Heart Failure, *SENIORS* Study of Effects of Nebivolol Intervention on Outcomes and Rehospitalization in Seniors with Heart Failure, *TOPCAT* Treatment of Preserved Cardiac Function Heart Failure with an Aldosterone Antagonist, *EMPHASIS-HF* Eplerenone in Mild Patients Hospitalization and Survival Study in Heart Failure, *SGLT2* sodium glucose cotransporter 2, *EMPEROR* Empagliflozin Outcome Trial in Patients with Chronic Heart Failure, *DAPA-HF* Dapagliflozin and Prevention of Adverse Outcomes in Heart Failure, *DELIVER* Dapagliflozin Evaluation to Improve the Lives of Patients with Preserved Ejection Fraction Heart Failure, *PRAISE-2* Prospective Randomized Amlodipine Survival Evaluation-2, *VICTORIA* Vericiguat Global Study in Subjects with Heart Failure with Reduced Ejection Fraction

Given that certain agents (such as metoprolol, carvedilol, and MRAs) without clear evidence of BP-lowering effect, significantly improved outcomes in HFrEF [70, 83] and that some agents (such as calcium channel blockers [CCBs], moxonidine, and α-blockers) with significant BP-lowering effects in the general hypertensive population had no/harmful effects on HFrEF [84, 85], it is now established that lowering BP per se is not associated with improved outcomes in HF. Instead, the focus has shifted to the class of drugs and how early they are initiated, forming the foundation of the current HF pharmacotherapy with individualized combination therapy in addition to existing agents. In this regard, patients with HF who have low BP are often undertreated, and as the Change the Management of Patients with Heart Failure (CHAMP-HF) registry data show, low BP is an independent predictor of the underuse or underdosing of neurohormonal antagonists [86]. Emphasizing that in certain cases, optimizing GDMT can be advantageous when patient tolerance permits, rather than refraining from medication solely due to BP concerns remains crucial.

## Treatment for BP in patients with HF

### Management of BP for incident HF

Recognizing the explicit risk of cardiovascular disease progression in patients with uncontrolled BP, considering hypertension as a precursor to HF remains crucial. The Staging Classification of Heart Failure (A, B, C, D), introduced by the American College of Cardiology/American Heart Association in 2003, highlights the preventive aspect of HF and underscores the significance of risk factor management [87, 88]. Accumulating evidence shows that that antihypertensive treatment is beneficial for incident HF. In a meta-analysis that demonstrated substantial reductions in cardiovascular death, stroke, and HF compared to placebo, the most significant benefit derived from antihypertensive therapy was the prevention of HF [89]. This analysis included 42 clinical trials with a total of 192,478 randomized patients and showed that low-dose diuretics significantly reduced the risk of stroke, cardiovascular mortality, and total mortality compared to placebo, with relative risks of 0.71, 0.81, and 0.90, respectively. The greatest reduction was observed in the risk of HF (relative risk, 0.51; 95% confidence interval, 0.42–0.62). More specifically, another meta-analysis by Ettehad et al. [90] showed that for each 10-mmHg reduction in SBP, the risk of HF significantly decreased by 28%.

In Hypertension in the Very Elderly Trial (HYVET) study, active antihypertensive treatment with indapamide, with or without perindopril, reduced the risk of incident HF by 64% in patients aged ≥80 years [91]. When comparing BP after 2 years of treatment, BP reduction was more modest in the perindopril group than that in the placebo group, with an additional reduction in SBP/DBP of 15.0/6.1 mmHg. In addition to placebo-controlled trials, several studies comparing active treatment with standard treatment for hypertension have reported data on the incidence of HF. The Systolic Blood Pressure Intervention Trial (SPRINT), which assessed the role of intensive antihypertensive treatment with a target SBP < 120 mmHg, showed a 38% reduction of relative risk in the development of HF in the intensive treatment group [57].

Despite increasing evidence highlighting the significant burden of HF associated with hypertensive heart disease, current hypertension treatment guidelines lack specific pharmacological strategies for managing patients beyond BP reduction [80, 81]. However, a position paper by the Heart Failure Association, in collaboration with the European Association of Preventive Cardiology, suggests utilizing diuretics, ACEis, and ARBs to prevent HF in hypertensive patients [92]. This recommendation is based on a network meta-analysis encompassing 26 trials, which showed that these three classes of antihypertensive drugs were most effective in lowering the incidence of HF compared to placebo. Furthermore, the 2023 European Society of Hypertension guidelines recommended lowering BP with five major antihypertensive drugs including CCBs and β-blockers, in addition to the above three classes of drugs, to prevent HF development [93]. In addition, if the target blood pressure is not achieved with these medications alone, additional medications (e.g., α-blockers) are recommended as needed.

### Management of BP in established HF

For patients with established HF, the prognostic meaning of BP is relatively different. Given that many HF drugs have BP-lowering effects, and that BP is one of the most important hemodynamic markers in cardiovascular disease and one of the few that can be measured directly in the clinic, BP targeting in HF is always of interest. However, there is no compelling evidence or guidelines on this aspect. Interestingly, standard HF therapy (with ACEi/ARBs, ARNIs, and β-blockers) may induce hypotension, occasionally leading to drug discontinuation. However, current HF guidelines recommend uptitrating medications to the tolerance of patients and emphasize that repeated attempts at uptitration can result in optimization, even if the initial attempts may fail [9, 94]. This is a substantial challenge and a gap between the ideal and the reality frequently encountered in clinical practice. The following questions arise: Should we aggressively pursue different classes of HF medications, even in those who have low BP, high frailty, and especially, intolerance to BP-lowering medications? Alternatively, should we maintain a certain target BP, for example, an SBP between 110 and 130 mmHg, even if it means discontinuing certain medications? The answers to these questions can be estimated through previous literature, and we should at least attempt to learn from existing evidence.

Recommendations on BP in the treatment of HF from several guidelines for HF and hypertension are summarized in Table 3 [9, 38, 81, 93, 95, 97, 98]. The 2021 European Society of Cardiology HF guideline emphasizes striving to achieve target dose of each HF medication, and the 2023 European Society of Hypertension guideline recommends combining the medications (ACEis [ARBs if not tolerated], ARNIs, BBs, MRAs, and SGLT2 inhibitors) that have been shown to have outcome benefits, particularly in HFrEF. It was common across guidelines that nondihydropyridine CCB agents were not recommended in HFrEF.

**Table 3:** Summary of recommendations for BP management in patients with HF

HF guideline	HFrEF	HFpEF
2016 ESC Heart Failure guidelines [38]	BP targets recommended in general population are applicable for HF.	
	First line: ACEi/ARB, BB, and MRA Second line: thiazide and loop diuretics Avoid nondihydropyridine, CCB, and moxonidine.	Nondihydropyridine CCBs are believed to be safe for HFpEF.
2021 ESC Heart Failure guidelines [95]	BP targets are uncertain for both HFrEF and HFpEF.	
	Every effort should be made to reach target doses of evidence-based medications, despite slight hypotension.	Patients with LVH and limited preload reserve, hypotension should be avoided.
2017 AHA Heart Failure guidelines [81]	In patients at an increased risk (stage A) for HF (estimated risk of cardiovascular disease ≥10%), the optimal target BP is 130/80 mmHg [96].	
	Target SBP < 130 mmHg (stage C, threshold is not yet proven in RCTs for the HF population)	If BP is high even after adequate volume control, target SBP < 130 mmHg (stage C).
2022 AHA Heart Failure guidelines [9]	In patients with hypertension (stage A), BP should be controlled to prevent symptomatic HF. No mention regarding target BP for stages B, C, and D.	
	CCB (dihydropyridine and nondihydropyridine) is not recommended.	BP targets are extrapolated from the trials for hypertension management in general. However, the optimal target or regimens are not known.
2022 Korean Society of Heart Failure guidelines [97]	Regardless of LVEF, if BP is > 140/90 mmHg, antihypertensive treatment may be helpful. If LVH is present, a combination of RAS inhibitors with CCBs (dihydropyridine) and/or diuretics is recommended, and is helpful to reduce SBP to the range of 120–130 mmHg.	
	The use of ACEi/ARBs, BBs, diuretics, and MRAs is recommended for BP management.	Target BP is reasonably set to a similar level as that of patients with HFrEF.
2023 ESH hypertension guidelines [93]	Combination of drugs with documented outcome benefits; ACEis (ARBs if not tolerated), ARNIs, BBs, MRAs, and SGLT2 inhibitors. Nondihydropyridine CCB is not recommended.	All major antihypertensive drug classes (ACEis or ARBs, BBs, CCBs, and thiazide/thiazide-like diuretics) are recommended. SGLT2 inhibitors are recommended independent of type 2 diabetes. ARNIs and MRAs can be considered, particularly in the lower LVEF spectrum.
2022 KSH hypertension guidelines [98]	In patients with hypertension who are at high risk for HF or with HF, it is reasonable to control BP below 130/80 mmHg.	

*BP* blood pressure, *HF* heart failure, *HFrEF* heart failure with reduced ejection fraction, *HFpEF* heart failure with preserved ejection fraction, *ESC* European Society of Cardiology, *ACEi* angiotensin-converting enzyme inhibitor, *ARB* angiotensin receptor blocker, *BB* β-blocker, *MRA* mineralocorticoid receptor antagonist, *CCB* calcium channel blocker, *LVH* left ventricular hypertrophy, *AHA* American Heart Association, *RCT* randomized controlled trial, *LVEF* left ventricular ejection fraction, *RAS* renin-angiotensin system, *SBP* systolic blood pressure, *ESH* European Society of Hypertension, *ARNI* angiotensin receptor-neprilysin inhibitor, *SGLT2* sodium glucose cotransporter 2, *KSH* Korean Society of Hypertension

### Target BP in established HF

The 2017 American College of Cardiology Foundation/American Heart Association guidelines for the management of HF recommend that optimal BP in those with hypertension and an increased risk of HF (stage A) should be < 130/80 mmHg [81]. In addition, patients with HFrEF and hypertension should be treated by GDMT titration to attain a target SBP < 130 mmHg. The target BP was also updated based on several clinical trials, primarily the SPRINT trial [99]. The 2022 focused update of Korean Hypertension Society guideline for the management of hypertension also mentioned that in patients with hypertension who are at high risk for HF or with HF, it is reasonable to control BP below 130/80 mmHg [98]. However, thus far, there are no compelling data to identify a simple BP target in patients with established HF.

In 2022, the American Heart Association/American College of Cardiology/Heart Failure Society of America updated guidelines for the management of HF, which stated that the optimal BP or antihypertensive regimens are not known for HFpEF and did not mention any BP goals for HFrEF at all [100]. As more pharmacological options become available in the modern era, the recent trend is toward maximizing GDMT within a tolerable range rather than providing a target BP. Here, the tolerability of an individual to treatment is assessed using safety indicators such as hypotension or renal insufficiency. If there are no adverse events, maximizing GDMT is deemed more important, suggesting that clinicians should not passively treat by solely providing a target BP.

### Differences in BP management between those with HFrEF and HFpEF

In general, guideline-recommended BP management for HFpEF was not significantly different from that for HFrEF. The difference is that hypertension is not as prevalent in HFrEF as in HFpEF, and patients with HFrEF rarely have uncontrolled BP [95]. In hypertensive patients, CCB is an option for BP control, although as mentioned above, the role of CCBs in HFrEF is limited (Table 2). However, the role of CCBs in HFpEF in the current era is not necessarily associated with worse HF outcomes. Although the Prospective Randomized Amlodipine Survival Evaluation-2 (PRAISE-2) study, which did not show the efficacy of amlodipine in HFrEF, had limited baseline medical treatment to ACEi (99%) and β-blocker use (19%) [78], a recent observational study on CCBs in HFpEF showed the noninferiority of CCBs, both dihydropyridines and nondihydropyridines, in addition to multiple drug usage, with β-blocker being used in more than two-thirds and MRA in one-quarter of the cases [101]. Although randomized clinical studies are required, evidence from studies on HFpEF suggests that CCB may still be effective in lowering BP and improving outcomes. In other words, it suggests that more aggressive BP management is feasible and effective by utilizing conventional antihypertensive agents to improve outcomes in patients with HFpEF compared to those with HFrEF. The 2023 European Society of Hypertension guideline also mentioned that the use of all major antihypertensive drugs including CCBs are recommended in HFpEF, and the use of ARNIs or MRAs can be considered in HFpEF with lower LVEF spectrum (Table 3).

### Time in BP target range in HF

A practical limitation of what we learn from clinical research is that BP measurements are taken only at a certain point in time. BP is a continuous metric that changes over time, so continuous BP monitoring and “time in target range” is also important for BP management, and some recent studies reinforce this point of view. Huang et al. [102] reported a post hoc analysis of the Treatment of Preserved Cardiac Function HF with an Aldosterone Antagonist (TOPCAT) trial, which compared the efficacy of spironolactone in patients with HFpEF and showed that the duration in the target range of SBP between 110 and 130 mmHg was associated with better clinical outcomes, including mortality and hospitalizations for HF. Moreover, subgroup analyses showed that it was more significant in younger patients than in older patients.

In addition, Chen et al. [103] reported another post hoc analysis of data from the TOPCAT trial and the Beta-Blocker Evaluation of Survival Trial (BEST), which showed that a longer duration of BP in the target range of SBP between 120 and 130 mmHg was associated with a lower risk of major adverse cardiovascular events in hypertensive patients with HF. Since the BEST trial enrolled patients with HFrEF and the TOPCAT trial enrolled patients with HFpEF, this post hoc study concluded that a longer duration in the target range was highly associated with better cardiovascular outcomes regardless of LVEF. However, these studies were still limited by the fact that they did not analyze different combinations of various HF drug classes. Therefore, additional studies with similar designs are anticipated to provide additional insights into BP management in the HFrEF population.

## Conclusions

Most of the HF medications have a mechanism and effect of lowering BP. Addressing patients with marginal BP poses significant therapeutic challenges, particularly considering that several other medications or clinical situations can also lower BP. Given the association of low BP with adverse prognosis, establishing a target BP and determining the ideal treatment strategy are critical, yet complex.

Many of these questions remain unanswered. How do we set a target BP? Can we unify all patients with HF using a single target BP? How do we individualize treatment and divide that subgroup? What evidence should we base our treatment on, and how do we categorize these patients for clinical research? How do we identify those who can benefit from further BP reduction and those who cannot? Which of the various HF medications should be titrated first for BP, when, and how much? Determining the optimal timing, dosage adjustments, and titration strategies for HF medication in the context of BP management requires further investigation.

Furthermore, the target BP varies depending on factors such as the patient’s condition, underlying comorbidities, etiology of HF, and the response of BP to medications. Some individuals have preserved tissue perfusion and no symptoms or signs of exercise intolerance or organ hypoperfusion even at lower BP, whereas others develop these dysfunctions even at normal or high BP. This highlights the difficulty of adopting a one-size-fits-all approach for treating HF, and it is hoped that more targeted treatments will become available depending on the underlying pathogenesis of HF.

## Data Availability

Not applicable.

## Abbreviations

ACEi: Angiotensin-converting enzyme inhibitor
AHA: American Heart Association
AHF: Acute heart failure
ARB: Angiotensin receptor blocker
ARNI: Angiotensin receptor-neprilysin inhibitor
BB: β-blocker
BEST: Beta-blocker Evaluation of Survival Trial
BP: Blood pressure
CCB: Calcium channel blocker
CHAMP-HF: Change the Management of Patients with Heart Failure
CHARM: Candesartan in Heart failure Assessment of Reduction in Mortality and Morbidity
CI: Confidence interval
COPERNICUS: Carvedilol Prospective Randomized Cumulative Survival
CV: Cardiovascular
DAPA-HF: Dapagliflozin and Prevention of Adverse outcomes in Heart Failure
DBP: Diastolic blood pressure
DELIVER: Dapagliflozin Evaluation to Improve the Lives of patients with Preserved Ejection Fraction Feart Failure
DIG: Digitalis Investigation Group
EMPEROR: Empagliflozin Outcome Trial in patients with Chronic Heart Failure
EMPHASIS-HF: Eplerenone in Mild Patients Hospitalization and Survival Study in Heart Failure
ESC: European Society of Cardiology
ESH: European Society of Hypertension
GDMT: Guideline-Directed Medication Therapy
H-AHF: Hypertensive acute heart failure
HF: Heart failure
HFmrEF: Heart failure with mildly reduced ejection fraction
HFpEF: Heart failure with preserved ejection fraction
HFrEF: Heart failure with reduced ejection fraction
HHF: Hospitalization for heart failure
HR: Hazard ratio
HYVET: Hypertension in the Very Elderly Trial
I-PRESERVE: Irbesartan in heart failure with Preserved ejection fraction
KorAHF: Korean Acute Heart Failure
KSH: Korean Society of Hypertension
LV: Left ventricular
LVEF: Left ventricular ejection fraction
LVH: Left ventricular hypertrophy
MERIT-HF: Metoprolol CR/XL Randomized Intervention Trial in Congestive Heart Failure
MI: Myocardial infarction
MRA: Mineralocorticoid receptor antagonist
NA: Not applicable
NYHA: New York Heart Association
OPTIMIZE-HF: Organized Program to Initiate Lifesaving Treatment In Hospitalized patients with Heart Failure
PARADIGM-HF: Prospective Comparison of ARNI with ACEi to determine Impact on Global Mortality and Morbidity in Heart Failure
PARAGON-HF: Prospective Comparison of ARNI with ARB Global Outcomes in Heart Failure with preserved ejection fraction
PEP-CHF: Perindopril in Elderly People with Chronic Heart Failure
PRAISE-2: Prospective Randomized Amlodipine Survival Evaluation-2
PRECISE: Prospective Randomized Trial of the Optimal Evaluation of Cardiac Symptoms and Revascularization
RAS: Renin-angiotensin system
RCT: Randomized controlled trial
SBP: Systolic blood pressure
SENIORS: Study of Effects of Nebivolol Intervention on Outcomes and Rehospitalization in Seniors with Heart Failure
SGLT2: Sodium glucose cotransporter 2
SOLVD: Studies Of Left Ventricular Dysfunction
SPRINT: Systolic Blood Pressure Intervention Trial
TNT: Treating to new targets
TOPCAT: Treatment Of Preserved Cardiac Function Heart Failure with an Aldosterone Antagonist
Val-HeFT: Valsartan-Heart Failure Trial
VALUE: Valsartan Antihypertensive Long-term Use Evaluation
VICTORIA: Vericiguat Global study in subjects with Heart Failure with Reduced Ejection Fraction
